# Single-cell transcriptome profiling reveals several LncRNAs differentially expressed in idiopathic germ cell aplasia

**DOI:** 10.3389/fcell.2022.952518

**Published:** 2022-09-06

**Authors:** Giovanni Lavorgna, Anna Sofia Tascini, Alessandro Bertini, Francesco Lanzaro, Francesco Montorsi, Massimo Alfano, Andrea Salonia

**Affiliations:** ^1^ Division of Experimental Oncology/Unit of Urology, URI, IRCCS Ospedale San Raffaele, Milan, Italy; ^2^ Center for Omics Sciences, IRCCS Ospedale San Raffaele, Milan, Italy; ^3^ University Vita-Salute San Raffaele, Milan, Italy

**Keywords:** LncRNAs, idiopathic germ cell aplasia, fertility, testes, obesity, TAF9B, bisphenol A, MEG3

## Abstract

Mechanisms underlying severe male infertility are still largely elusive. However, recently, a single-cell transcription study by our group identified several differentially expressed coding genes in all the somatic cell types in testes of patients with idiopathic germ cell aplasia (iGCA). Here, we leverage this work by extending the analysis also to the non-coding portion of the genome. As a result, we found that 43 LncRNAs were differentially expressed in the somatic cells of these patients. Interestingly, a significant portion of the overexpressed LncRNAs was found to be a target of TAF9B, a transcription factor known to be involved in germ cell survival. Moreover, several overexpressed LncRNAs were also found to be activated in a mouse model of Sertoli cells treated with bisphenol A, a widespread environmental contaminant, long suspected to impair male fertility. Finally, a literature search for MEG3, a maternally imprinted LncRNA overexpressed as well in our patients, found it to be involved, among other things, in obesity and inflammation, known comorbidities of iGCA, ultimately suggesting that our findings deepen the understanding of the molecular insights coupled not only to the pathogenesis, but also to the clinical course of this class of patients.

## Introduction

The successful completion of spermatogenesis relies on the availability of spermatogonial stem cells, along with their capability to proliferate and to transform, first, into progenitor spermatogonia and, then, into spermatozoa (Oatley and Brinster, 2008). Sertoli-cell-only (SCO) syndrome, also known as germ cell aplasia (GCA), represents a condition of the testes where only Sertoli cells line the seminiferous tubules, ultimately leading to the most severe form of male infertility, the non-obstructive azoospermia (NOA) (Ramphul and Mejias, 2021).

While a genetic component has been determined for NOA (Nakamura et al., 2017), it is becoming clear that, in the vast majority of cases, there is not an evident underlying cause, leading to the broad definition of those cases as the wide group idiopathic NOA (iNOA) (European Association of Urology, 2022). Instead, there is overwhelming evidence that the male infertility status, and mainly in NOA cases, is linked with an augmented risk of diseases associated with aging, like type II diabetes, cardiovascular disease, autoimmune disease, obesity and cancers (Eisenberg et al., 2015). All this has brought to the development of a new conceptualization of the male fertility status as a proxy of the overall men’s health (Salonia et al., 2009).

Recently, single-cell RNA-sequencing (scRNA-seq) analysis from our group identified eight cell clusters in the testicular somatic cells populations of iGCA patients (Alfano et al., 2021). Thanks to the use of cell type marker genes on these clusters, the main somatic cell populations were recognized, Leydig (LEY), myoid (MYD), Sertoli (SRT) and endothelial (END) cells. Moreover, immune cells were also recognized, like macrophages (MCR) and T-cells (TCL). Finally, the stromal (STRO) cluster was assigned to pericytes or vascular smooth muscle cells, whereas one cluster, lacking clear marker genes, remained undetermined (UND). Identification of DE (DE) coding genes, lead to the identification of molecular pathways related to aging, inflammation and DNA damage, offering molecular insights into the pathogenesis of iGCA.

In the present study, we dissected the dysregulation of the non-coding transcripts in iGCA by focussing our attention to the analysis of the LncRNAs of our dataset. As a result, we found 43 DE LncRNAs in iGCA patients. Our analysis revealed a close connection between differential LncRNAs and several features of this disease, such as the survival of germ cells and the presence of comorbidities, like obesity and inflammation. Also, an environmental contribution to the disease insurgence was found to be highly consistent with our data.

## Methods

### Identification of DE LncRNAs

**FIGURE 1:**
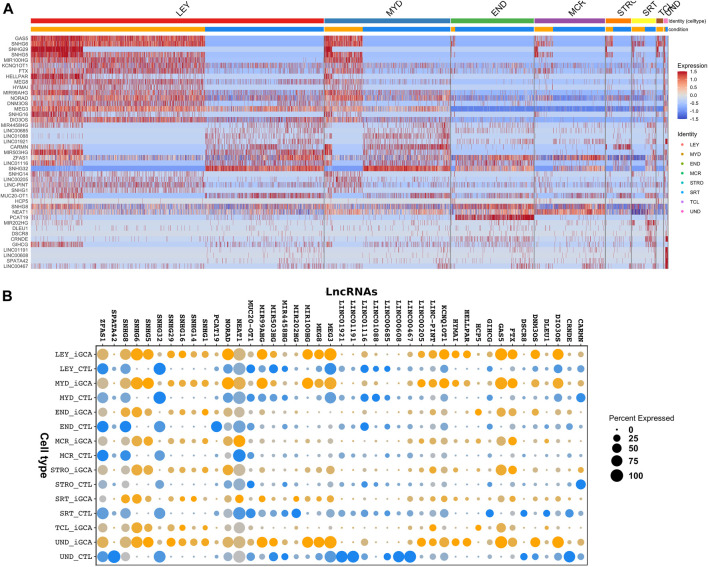
Differential expression of 43 LncRNAs in iGCA patients. **(A)** Heatmap showing the quantification of the differentially expressed LncRNAs in each cell type in patients (orange horizontal bar) vs. healthy controls (blue horizontal bar). **(B)** Dot plot comparison of DE LncRNAs. Dot color intensity is proportional to the average expression level for each group and the dot size is related to the percentage of expressing samples. Indicated cell types are: Leydig (LEY), myoid (MYD), endothelial (END) cells, macrophages (MCR), stromal (STRO) and Sertoli (SRT) cells, T-cells (TCL), undetermined (UND).

Tissue processing, ethical approval and the scRNA-seq procedure were already described (Alfano et al., 2021). DE LncRNAs in iGCA patients of this study were identified by mining DE transcript names in Supplementary Dataset 29 for the presence of the term “lncRNA” in the “gene_type” field of the “gencode.v39. annotation.gtf” file from the GENCODE project (www.gencodegenes.org). Normalized transcript expression data were extracted and used to build the heat map and the dot plot shown, respectively, in Figure 1A and Figure 1B. Normalized expression counts, along with pertinent sample metadata, are available as Supplementary Data respectively in the files “Normalized_LncRNA_counts.csv” and “Sample-phenotypes.csv”. Upstream and downstream transcripts of the DE LncRNAs located within 500 Kb of the beginning of each locus were obtained from the ‘gencode.v39. annotation.gtf’ file.

### Literature search

In order to evaluate the consistency of the MEG3 role in (IR) injury, we performed a literature search on 11 February 2022 using the keywords “MEG3 AND Reperfusion Injury”, “MEG3 AND infarction” and “MEG3 AND organ failure”. Both titles and abstracts were retrieved and, whenever it deemed to be necessary, also the full article was analyzed. A similar search strategy was used for evaluating the correlation between MEG3 expression and obesity/diabetes with Pubmed abstracts being retrieved using the keywords “MEG3 AND obesity” and “MEG3 AND diabetes”. Statistical significance was calculated by using a binomial test.

### Identification of signatures associated to DE LncRNAs

All the datasets (C1—> C8 plus the hallmark gene set) from the MSigDB 7.5.1 database (Liberzon et al., 2015) were searched for overlaps with our DE LncRNAs using the “Investigate Gene Set” option with a FDR of 0.001. A custom R script was used to analyze a non-redundant, 147 libraries dataset available at the Enrichr resource (Xie et al., 2021) through the CRAN library “enrichr”. Differential expression of the LncRNAs was measured using the “FindMarkers” program from the R Seurat software package (Satija et al., 2015), using default parameters (Alfano et al., 2021). Differerential expression for the TAF9B transcript in the LEY and MYD cell types was measured using the ‘FindMarkers’ program as well, except that the “logfc.threshold” parameter, controlling the minimum differential expression fold change cutoff to be used between the two groups of cells, was set to zero instead of the default value of 0.25.

## Results

### Identification of DE LncRNAs in idiopathic germ cell aplasia patients

We searched our previously identified (Alfano et al., 2021) dataset of DE transcripts in iGCA patients for the presence of LncRNAs. This dataset profiled, by means of scRNA-seq, gene expression in each somatic cell type in testes of men with iGCA (Alfano et al., 2021). As a result, 43 LncRNAs were found to be DE in one or more cell types, as shown in the heatmap in Figure 1A and in Table 1. Further details on their genomic localization and on their differential expression are reported, respectively, in Supplementary Table S1 and in the dot plot comparison in Figure 1B, with the dot color intensity proportional to the average expression level for each group and the dot size related to the percentage of expressing samples. Among these transcripts, differential expression was very consistent, with 21 LncRNAs being downregulated in one or more cell types and 21 LncRNAs being instead upregulated in one or more cell types. Only the LncRNA NEAT1 presented a mixed expression, being downregulated in endothelial cells and upregulated in Sertoli cells (Figure 1B and Table 1).

**TABLE 1:** LncRNAs differentially expressed in idiopathic germ cell aplasia (iGCA) patients. The number of cell types showing differential expression is reported, along with the direction of expression (“UP” or “DOWN”) and the cell type where differential expression occurs.

1	SNHG14	UP	MYD
1	HCP5	UP	END
1	SNHG16	UP	LEY
2	HYMAI	UP	LEY, MYD
2	MIR99AHG	UP	LEY, MYD
2	DIO3OS	UP	LEY, MYD
2	DNM3OS	UP	LEY, MYD
2	MEG8	UP	LEY, MYD
2	SNHG1	UP	MYD, END
2	KCNQ1OT1	UP	LEY, MYD
3	MIR100HG	UP	LEY, MYD, UND
3	FTX	UP	LEY, MYD, STRO
3	NORAD	UP	LEY, MYD, MCR
3	MEG3	UP	LEY, MYD, STRO
4	SNHG29	UP	LEY, MYD, MCR, STRO
7	SNHG5	UP	LEY, MYD, END, MCR, STRO, SRT, UND
7	SNHG6	UP	LEY, MYD, END, MCR, STRO, SRT, UND
7	GAS5	UP	LEY, MYD, END, MCR, STRO, SRT, UND
2	NEAT1	UP/DOWN	END, SRT

### Enrichment of DE LncRNAs in biological signatures

To evaluate the enrichment of the DE LncRNAs in specific lists or biological signatures, all the datasets from the Molecular Signature Database (Liberzon et al., 2015) were firstly searched. Since some of our cell types were not amenable to meaningful searches because they contained only a few DE LncRNAs, like for example STRO cells possessing only three downregulated LncRNAs (see Table 1), a pool of all cell types was used in our searches. To compensate for this added heterogeneity, a rather highly stringent FDR threshold, 0.001, was used. As a result, while no significant enrichment was found in the downregulated LncRNAs, the overexpressed ones were found to be significantly enriched in chromatin immunoprecipitation targets of the TATA-Box Binding Protein Associated Factor 9b (TAF9B), a basal component of the nuclear transcription machinery (Frontini et al., 2005), as shown in Table 2. Previous analysis (Alfano et al., 2021) was unable to detect any upregulation of TAF9B in cell types overexpressing a significant fraction of its target LncRNAs: LEY (6 target LncRNAs) and MYD (7 target LncRNAs). However, this analysis was performed using a relatively large log fold change (FC) cutoff, 0.25. Since for regulatory molecules, like TAF9B, even smaller FC can have a great significance in high throughput, transcriptional profiling experiments (Dalman et al., 2012), we repeated the analysis without employing this cutoff. As a result, it was found that TAF9B was significantly overexpressed, albeit with a lower log FC, both in LEY and MYD cell types, as shown in Supplementary Table S2, ultimately supporting its potential role as a transcriptional activator of DE LncRNAs in iGCA.We also looked for enrichments of our DE LncRNAs in the several datasets available at the Enrichr resource (Xie et al., 2021). Also in this case, no hit was found for the downregulated ones, but, as shown in Table 2, a highly significant enrichment was found with the genes overexpressed in a mouse model of Sertoli cells treated with Bisphenol A (BPA) (Tabuchi et al., 2006), a compound employed in the preparation of various plastics and known to be an endocrine disruptor, exhibiting weak estrogenic, anti-thyroid and anti-androgenic activities (Peretz et al., 2014). Similar results were obtained using single, i.e., no pooled, cell types harboring a high number of DE LncRNAs, like LEY (*n* = 16) and MYD (*n* = 18) cells (Supplementary Table S3).

**TABLE 2:** Significative enrichments in public datasets of the differentially expressed LncRNAs i pooled cell types.

Source	Gene Set Name	Overlap	p-value	FDR q-value	Hits
C3 (MsigDB)	TAF9B_TARGET_GENES	8/569	3.68E-10	1.21E-05	FTX;GAS5;KCNQ1OT1;NEAT1;NORAD;SNHG1;SNHG5;SNHG6
Drug_Perturbations_from_GEO_up	Bisphenol A 6623 mouse GSE4650 sample 3575	5/225	3.88E-06	9.67E-04	SNHG1;SNHG6;NEAT1;SNHG5;GAS5

### Literature survey and meta-analysis of the LncRNA MEG3

A bibliographical search, aimed at evaluating possible relationships of the DE LncRNAs to disease features, was also undertaken. We started our analysis with the MEG3 LncRNA because it had a relatively high number of abstracts available in Pubmed (#931, as of 11 February 2022). MEG3, overexpressed in LEY, MYD and STRO cell types of our patients, was an interesting molecule also because of its role as a maternally imprinted transcript (Schmidt et al., 2000). Moreover, given the relevant role played by inflammation in the testes of our patients (Alfano et al., 2021), it was i intriguing that, recently, MEG3 was found to promote pyroptosis, i.e., a form of cell-death associated with inflammatory signals, in testicular ischemia-reperfusion (IR) injury (Ning et al., 2021). In order to evaluate the consistency of the MEG3 role in IR injury, we performed a literature search using pertinent keywords (see Methods). A total of 29 experimental, non-redundant articles were retrieved and the direction of MEG expression (up or down) was evaluated. Results, shown in Table 3, indicated that MEG3 expression was upregulated in all cases, strongly validating it as a potential therapeutic target in our patients. Given the relevant role of the body-mass index (BMI), obesity and diabetes as comorbidities in iGCA (Boeri et al., 2022), we also performed a literature search in order to determine MEG3 direction of expression in these circumstances. Also in this case, albeit not exclusively, MEG3 upregulation was found to be correlated to these diseases (*p* = 0.02), as shown in Supplementary Table S4.

**TABLE 3:** MEG3 expression direction in Pubmed articles related to IR Injury, infarction and organ failures.

Reference	DOI	MEG3	Organ/Tissue	Disease/Condition	CellularModel	AnimalModel	ClinicalSamples
Ding, H et al. (2020)	10.21037/jtd-19-2472	UP	Aorta	IRI via chronic intermittent hypoxia	NA	Mouse	NA
Liang, J et al., 2020	10.1016/j.expneurol.2019.113139	UP	Brain	Cerebral ischemia-reperfusion	Neurocytes	Rat	NA
Zhou, X et al., 2018	10.1002/jcb.28075	UP	Brain	Hypoxic-ischemic brain damage	NA	Mouse	NA
Zhan, R et al., 2017	10.1016/j.bbrc.2017.06.104	UP	Brain	Oxygen-glucose deprivation/reoxygenation	Rat endothelial cells	NA	NA
Yan, H et al., 2017	10.1038/s41419-017-0047-y	UP	Brain	Ischemic stroke	N2a	Mouse	NA
Yan, H et al., 2016	10.1016/j.neuroscience.2016.09.017	UP	Brain	Ischemic neuronal death in stroke	NA	Mouse	NA
Luo, H et al., 2020	10.1074/jbc.RA119.010946	UP	Brain	Ischemic stroke	NA	Mouse	NA
Shen, J et al., 2018	10.1080/21691401.2018.1471483	UP	Brain	Cerebral infarction	NA	Rat	NA
Deng, D et al., 2020	10.1080/21691401.2020.1725533	UP	Brain	Hypoxic-ischaemic brain damage	PC12	NA	NA
Xiang, Y et al., 2020	10.18632/aging.102790	UP	Brain	Ischemic stroke	N2a	Mouse	NA
Li, T et al., 2020	10.1152/japplphysiol.00433.2020	UP	Brain	Polarization of microglia in cerebral IR injury	NA	Mouse	NA
Xie, B et al., 2021	10.12659/MSM.929435	UP	Brain	Intracerebral hemorrage	NA	Rat	NA
You, D et al., 2019	10.1016/j.biopha.2018.12.067	UP	Brain	Cerebral ischaemia riperfusion injury	NA	Rat	NA
Liu, X et al., 2016	10.3389/fncel.2016.00201	UP	Brain	Neuron apoptosis by hypoxia	HT22	Mouse	NA
Chen, C et al., 2021	10.4081/ejh.2021.3224	UP	Brain	Ferroptosis	NA	Rat	NA
Zhou, Y et al., 2021	10.3892/mmr.2020.11656	UP	Heart	Hypoxiainduced injury in rat cardiomyocytes	H9c2	Rat	NA
Wu, H et al., 2018	10.1038/s41434-018-0045-4	UP	Heart	Myocardial infarction	NA	Mouse	Heart failure
Zhang, J et al., 2019	10.1038/s41598-018-36369-1	UP	Heart	Cardiac hypertrophy	Cardiomyocytes	Mouse	NA
Jinwen Su et al., 2018	10.1093/abbs/gmy133	UP	Heart	Hypoxic cardiac progenitor cells	Cardiomyocytes	NA	NA
Li, X et al., 2019	10.1111/jcmm.14714	UP	Heart	Myocardial infarction, hypoxic cardiomycytes	Cardiomyocytes	Mouse	NA
Xue, Y et al., 2020	10.1111/jcmm.15720	UP	Heart	Viral myocarditis	Cardiac tissue macrophages	Mouse	NA
Piccoli, M et al., 2017	10.1161/CIRCRESAHA.117.310624	UP	Heart	Cardiac remodelling	Cardiac fibroblasts	NA	NA
Li, W et al., 2021	10.3892/etm.2021.10704	UP	Heart	Hyperhomocysteinemia cardiac fibrosis	Cardiac fibroblasts	NA	NA
Liu, D et al., 2021	10.1038/s41419-021-03466-5	UP	Kidney	IRI	HK-2	Mouse	NA
Mao, H et al., 2021	10.1002/jbt.22649	UP	Kidney	Hypoxia/reoxygenation induced apoptosis	HK-2	NA	NA
Deng, J et al., 2021	10.3389/fphys.2021.663216	UP	Kidney	LPS-Induced Acute Kidney Injury	Renal tubular epithelial cells	Mouse	NA
Yang, R et al., 2018	10.1002/jcb.27163	UP	Kidney	LPS-induced apoptosis in renal tubular epithelial cells	TKPTS	Mouse	NA
Zou, D et al., 2020	10.1016/j.ajpath.2019.12.013	UP	Lung	Hyperoxia-Induced Lung Injury	NA	Mouse	NA
Ning, J et al., 2021	10.3389/fcell.2021.671613	UP	Testis	Testicular torsion	GC-1	Mouse	NA

IRI, Ischemia Reperfusion Injury; LPS, Lipopolysaccharide.

## Discussion

Here, we present a follow-up study to our previous characterization of the transcriptional atlas of the testicular somatic cell populations linked to human iGCA (Alfano et al., 2021). Previous work had indeed determined the contribution of several coding genes to the pathological status of these patients, underlying their relevance for frequently observed features in iGCA, like senescence of the testicular somatic cells, immaturity of LEY cells, persistent inflammation, DNA damage and defects in gene imprinting. The current work extends the previous analysis by looking also at the non-coding portion of the genome. While the inevitably low depth of the scRNA seq approaches somehow limits the identification of weakly expressed molecules like ncRNAs (Pokhilko et al., 2021), we still were able to identify 43 LncRNAs DE in iGCA patients. Also, in light of the extensive spectrum of comorbidities observed in unfertile males (Salonia et al., 2009; Shiraishi and Matsuyama, 2018; Boeri et al., 2022), the study of regulatory transcripts, like LncRNAs, in this class of patients appears to be of paramount importance.

Overexpressed LncRNAs in our patients were found to overlap a small, but biologically very relevant number of lists. For instance, the overlap with the target genes of TAF9B, a component of TFIID upregulated during the germline development (Gura et al., 2020), is rather intriguing. Indeed, it is in keeping with recent findings showing that cells of the germline use a highly specialized form of TFIID, the transcription initiation complex of nuclear genes that once was thought to be, instead, rather general and ubiquitous in all tissues (Hoey et al., 1990). Interestingly, TAF9B is also within the network of proteins regulated by the gene Deleted in Azoospermia Like (DAZL), whose function is essential for spermatogenesis and meiosis completion (Zagore et al., 2018; Li et al., 2019). Summing up, while the formal proof of the transcriptional activation of the identified LncRNAs by TAF9B must await further studies, present data are already suggestive of an involvement of this transcription factor in iGCA.

Of interest also appears the overlap with the transcripts stimulated by BPA exposure in mouse Sertoli cells, since the impact of the environment on reproductive health has been reported as an explanation for idiopathic infertility (Shi et al., 2017). It will be interesting to see if BPA can induce also the expression of the identified LncRNAs in human testicular somatic cells in *ex-vivo* experiments. BPA, along with its analogs, is present in several daily use products, like manufactured plastics, cans and paper (Wong and Durrani, 2017). It is known to possess a variety of deleterious effects on human health, including male reproduction and fertility (Siracusa et al., 2018). A replacement of BPA with more chemically stable analogs has been attempted in the manufacturing process. However, these analogs have proved to be both worse in terms of biodegradability and better in terms of dermal penetration (Liao et al., 2012). BPA, along with its analogs, contaminate the environment, including air, water, food and house dust (Wu et al., 2018). Therefore, the primary intake of bisphenols in humans is likely to occur through the diet, mainly through the consumption of canned foods or drinking bottled water (Eladak et al., 2015). All this ultimately suggests a limitation of their use, also in consideration of the wide spectrum of comorbidities being associated to BPA usage (Ma et al., 2019; Barra et al., 2022), even through the maternal route (Blaauwendraad et al., 2022).

A literature survey also found MEG3 consistently linked to IR injury and obesity/diabetes, validating it, or molecules in its pathway, as a therapeutic target in IGCA. MEG3 contribution to the inflammatory phenomena of this disease could be, indeed, rather important. Recently, inflammation induced by lipopolysaccharides (LPS) in Leydig cells was found to decrease both testosterone levels and cell viability, along with a MEG3 transcriptional activation (Zhou et al., 2021). Ablation of MEG3 expression in this setting attenuated the inflammatory response and increased testosterone levels through the sponging effect on miR-93-5p (Zhou et al., 2021). The sponging capabilities of MEG3, mainly related to the RI phenomenon, have been shown also in several other works (reviewed in Zhao et al., 2022). Therefore, miRNAs in its pathway are likely to be therapeutic targets as well in inflammatory phenomena and should be considered for therapeutic intervention.

## Data Availability

The original contributions presented in the study are included in the article/Supplementary Material, further inquiries can be directed to the corresponding authors.

## Supplementary material

Supplementary Table S1 Genomic coordinates of the 43 differentially expressed LncRNAs. The upstream and downstream transcripts located within 500 Kb are also reported.

Supplementary Table S2 TAF9B differential expression in iNOA vs. CTRL in LEY and MYD cell types.

Supplementary Table S3 Significative enrichments in public datasets of the differentially expressed LncRNAs in LEY and MYD.

Supplementary Table S4 MEG3 expression direction in Pubmed articles related to obesity and diabetes.
